# The IL-2 – IL-2 receptor pathway: Key to understanding multiple sclerosis

**DOI:** 10.1016/j.jtauto.2021.100123

**Published:** 2021-09-21

**Authors:** Daphne Peerlings, Max Mimpen, Jan Damoiseaux

**Affiliations:** a Central Diagnostic Laboratory, Maastricht University Medical Center, Maastricht, the Netherlands; b School for Mental Health and Neuroscience, University of Maastricht, Maastricht, the Netherlands

**Keywords:** Multiple sclerosis, Interleukin 2, Interleukin 2 receptor, Soluble interleukin 2 receptor

## Abstract

The development, progression, diagnosis and treatment of autoimmune diseases, such as multiple sclerosis (MS), are convoluted processes which remain incompletely understood. Multiple studies demonstrated that the interleukin (IL)-2 – IL-2 receptor (IL-2R) pathway plays a pivotal role within these processes. The most striking functions of the IL-2 – IL-2R pathway are the differential induction of autoimmune responses and tolerance. This paradoxical function of the IL-2 – IL-2R pathway may be an attractive therapeutic target for autoimmune diseases such as MS. However, the exact mechanisms that lead to autoimmunity or tolerance remain to be elucidated. Furthermore, another factor of this pathway, the soluble form of the IL-2R (sIL-2R), further complicates understanding the role of the IL-2 – IL-2R pathway in MS. The challenge is to unravel these mechanisms to prevent, diagnose and recover MS. In this review, first, the current knowledge of MS and the IL-2 – IL-2R pathway are summarized. Second, the key findings of the relation between the IL-2 – IL-2R pathway and MS have been highlighted. Eventually, this review may launch broad interest in the IL-2 – IL-2R pathway propelling further research in autoimmune diseases, including MS.

## Highlights

• The IL-2 – IL-2R pathway determines the balance between immunity and tolerance.
• The IL-2 – IL-2R pathway is involved in the pathogenesis of multiple sclerosis.
• The role of soluble IL-2R is controversial and requires further investigation.

## Abbreviations

CIS: Clinically isolated syndrome
CNS: Central nervous system
CSF: Cerebrospinal fluid
DCs: Dendritic cells
EAE: Experimental autoimmune encephalomyelitis
EDSS: Expanded disability status scale
GM-CSF: Granulocyte-macrophage colony stimulating factor
GWAS: Genome wide association studies
HLA: Human leukocyte antigen
IFN-γ: Interferon-γ
IL-2: Interleukin 2
IL-2R: Interleukin 2 receptor
LTα: lymphotoxin alpha
JAK: Janus tyrosine kinase
MAPK: Mitogen-activated protein kinase
MRI: Magnetic resonance imaging
MS: Multiple sclerosis
NK-cells: Natural killer cells
OND: Other neurological diseases
PI3K-AKT: Phosphoinositide-3-kinase-protein kinase B
PPMS: Primary progressive multiple sclerosis
pSTAT5: Signal transducer and activation of transcription 5 phosphorylation
RRMS: Relapsing and remitting multiple sclerosis
sIL-2R: Soluble interleukin 2 receptor
SPMS: Secondary progressive multiple sclerosis
STAT: Signal transducer and activation of transcription
T_c_: Cytotoxic T-cells
T_eff_: Effector T-cells
TNF-α: tumor necrosis factor alpha
TGF-β: transforming growth factor beta
T_h_: Helper T-cells
T_reg_: Regulatory T-cells
γ_c_-chain: Common γ-chain

## 1 Introduction

Multiple sclerosis (MS) is an inflammatory autoimmune disease affecting the central nervous system (CNS) [1]. Disturbances within immune-mediated processes cause loss of tolerance resulting in autoimmunity towards the CNS. The interleukin (IL-)2 – IL-2 receptor (IL-2R) pathway plays a paradoxical, but pivotal, role in the outcome of both immunity as well as tolerance and this is effectuated via multiple types of immune cells (*e.g.,* monocytes, macrophages, T-, B-, natural killer (NK)- and dendritic cells (DCs)) [2,3]. However, the underlying mechanisms that lead to these paradoxical outcomes remain to be elucidated. Understanding the role of the IL-2 – IL-2R pathway in autoimmune diseases like MS is further complicated by the existence of soluble (s)IL-2R, a receptor component most likely released by shedding from the cell surface of activated immune cells. The function of the sIL-2R is still speculative. In addition, previous studies demonstrated that genetic and environmental factors interact with the IL-2 – IL-2R pathway and may influence the final outcome [4,5]. For instance, vitamin D-deficiency is a well-known risk factor for MS and is linked to the cell-surface expression of the IL-2R [6]. The IL-2R has also been addressed as a therapeutic target by the introduction of daclizumab [[7], [8], [9]]. The working mechanism of this drug, unexpectedly, involved NK-cells and this has attracted novel attention for these cells in the complex pathogenesis of MS [10].

In order to illustrate the complexity of the IL-2 – IL-2R pathway in the balance between immunity and tolerance, this review further elaborates on this pathway in the context of MS. After a short introduction of the relevant clinical features of MS, the structure and the functions of the IL-2 – IL-2R pathway are described, with a focus on T- and NK-cells. Next, the current knowledge on potential roles of IL-2, IL-2R and, in particular, sIL-2R within MS are reviewed. Finally, in the discussion two examples are elaborated upon to show the interaction of the IL-2 – IL-2R pathway with environmental factors, *i.e.*, vitamin D, and therapeutic options, *i.e.*, daclizumab.

## 2 Multiple sclerosis

MS is a chronic inflammatory and autoimmune disease recognized by the degeneration and demyelination of neurons in the CNS. More than two million humans are affected by MS worldwide. Young female adults (20–40 years old) are two to three times more susceptible than young male adults, likely due to distinct endocrine-immune interactions [11,12].

The localization and extent of the inflammatory lesions in the CNS are linked to clinical symptoms and disease severity, respectively. These lesions are commonly seen in areas such as the optic nerve, brainstem, and cerebellum, predominantly in the white matter. As such, common symptoms of MS include blurred vision, sensory and motor disturbances, gait impairment, and imbalance [11,13]. The first presenting symptom induced by focal CNS demyelination is defined as clinically isolated syndrome (CIS). About 60–80% of individuals with CIS are eventually diagnosed with MS based on additional clinical manifestations [14]. The diagnosis of MS relies on the McDonald criteria 2017: imaging the dissemination of neurological lesions over space and time achieved by magnetic resonance imaging (MRI), cerebrospinal fluid analysis and neurological functional assays. The predominant diagnostic tool is MRI due to its high sensitivity for demonstrating the dissemination of lesions over space and time and discriminating MS from other MS-mimicking manifestations (*e.g.*, neuromyelitis optica spectrum disorder and acute disseminated encephalomyelitis). To provide supportive evidence of a MS diagnosis, cerebrospinal fluid (CSF) analysis may be performed by determining the white blood cell count, protein concentration and IgG oligoclonal bands [15].

The most common disease course in MS patients (85–90%) is the relapsing and remitting phenotype (RRMS). This is characterized by acute episodes, lasting at least 24 h, of neurological deficits with a sudden onset (defined as relapses). Every relapse is followed by a (partial) recovery phase where individuals remain neurologically stable (also referred to as remitting stage). About 60% of RRMS patients develop a secondary progressive stage (SPMS) where the clinical disability gradually progresses, in the absence of relapses. Some MS patients (10–15%), however, have from onset onwards a gradual progression of neurological dysfunction and never suffer from relapses and remissions, also known as primary progressive MS (PPMS) [14,16].

Understanding the disease course based on etiology and pathogenesis contributes to the prevention, diagnosis and treatment of MS. Although the exact etiology and pathogenesis is poorly understood, different factors have been discovered to play a role such as genetic and environmental sources [4]. It is believed that environmental (*e.g.*, virus infections, vitamin D deficiency, smoking and obesity) and genetic (*e.g.*, human leukocyte antigen (*HLA*) genes, and, among many other genes the *IL2RA* gene) factors may activate immune-mediated processes which play a major role in the pathogenesis of MS [5].

Due to previous research, it has been considered that autoreactive helper T-cells (T_h_; CD4^+^) are primarily driving the autoimmune reaction [17]. T_h_-cells, activated in peripheral lymph nodes, proliferate, express surface receptors and secrete pro-inflammatory molecules which eventually cross the blood-brain barrier. In the CNS, microglia, astrocytes and macrophages present autoantigens to reactivate these T_h_-cells via HLA class II. As discussed by Kunkl et al. (2020), several T_h_-cell subsets (*e.g.*, T_h_1, T_h_2, T_h_9, T_h_17 and T_h_22) have been associated with MS [18]. However, it remains speculative which is the most pathogenic T_h_-cell subset. Previously, it was thought that T_h_1-cells are the major pathogenic cells in MS since these cells and T_h_1-like cytokines (such as interferon-γ (IFN-γ) and IL-2), are abundantly present in spinal cord and brain lesions of experimental autoimmune encephalomyelitis (EAE) animals, an animal model that mimics MS [19]. This concept has been challenged due to the impact of IL-17 producing T_h_-cells, also suggested to represent T_h_17-cells. Cua et al. (2003) and Murphy et al. (2003) investigated the lack of IL-12 (p35−/−), IL-23 (p19−/−) and both cytokines (p40−/−) in autoimmune mice models (EAE and collagen-induced arthritis) [20,21]. IL-12 drives T_h_1 differentiation and is composed of the p35 and p40 subunits. IL-23 functions as a T_h_17 differentiation cytokine that consists of p19 and (the IL-12 shared) p40 subunits. Therefore, the deletion of p35−/−, p19−/− and p40−/− in mice resulted in the lack of T_h_1-cells, T_h_17-cells and both T_h_-cell subsets, respectively. In p35−/− mice lacking T_h_1-cells, EAE revealed an aggravated course, while p19−/− and p40−/− mice, both lacking T_h_17-cells, were resistant to induction of EAE. This indicates that T_h_17-cells, rather than T_h_1-cells, are crucial for development of EAE. Whether this essential role of T_h_17-cells also applies for MS remains a matter of discussion. In MS, dependent on the activated T_h_-cell subset, additional immune-members are recruited which contribute to MS-lesions. For instance, T_h_1-cells activate macrophages and cytotoxic T-cells (T_c_; CD8^+^). T_h_2-cells trigger B-cells to produce auto-antibodies against myelin sheaths, axons and oligodendrocytes. T_h_17-cells provoke the secretion of inflammatory proteins via macrophages, epithelial and endothelial cells and attract neutrophils to the inflammation [18]. However, to attain self-tolerance, regulatory T-cells (T_reg_) control these pathogenic responses of T_h_-cells. The function of T_reg_ is hampered in MS [22]. Another cell-type, the NK-cells, has also the capability to eliminate autoreactive T_h_-cells and, therefore, to suppress the pathogenic reactions. This was unraveled as the underlying mechanism of daclizumab, a monoclonal antibody targeting the IL-2Rα-chain (CD25) [8,9]. Overall, MS is a complex disease consisting of multiple components that either enable or prevent development of disease. Interestingly, the IL-2 – IL-2R pathway is involved in the effective function of the majority of these components, eventually deciding between autoimmunity and tolerance.

## 3 The IL-2 – IL-2R pathway

Since the discovery of IL-2 in 1975, this typical type 1 cytokine family member is recognized as a T-cell growth factor [23]. The cytokine of ∼15kD consists out of 4 α-helices. IL-2 is predominantly produced by activated T-cells (rather CD4^+^ than CD8^+^ cells) and to a lesser extent by B cells, NK-cells, NKT-cells, DCs and mast cells [[24], [25], [26], [27], [28]]. The activity of IL-2 is mediated via the IL-2R and can be elaborated in an autocrine and paracrine manner [29]. While IL-2 reactivity has been described for many different leukocyte subsets, the current review will focus on T-cells and NK-cells (Table 1).

**Table 1:** Primary IL-2 activities and IL-2R expression on different cell types.

**Cell types**	**Primary IL-2 activities**	**IL-2R expression**			**References**
		**α**	**βγ**	**αβγ**	
**CD4**^**+**^**T-cells**					
Naïve	- Not responsive	Low	Low	Low	(29, 105–108)
Activated	- Induces the proliferation and anti-apoptotic mechanisms of antigen-specific clones - Enhances (other) pro-inflammatory cytokine secretion (*e.g.,* IFN-γ, TNF-α, IL-4, IL-5 and IL-13) - Helper 1 and 2 T-cell differentiation - Inhibits pro-inflammatory cytokine secretion (*e.g.,* IL-17A and IL-22) - Suppresses helper 17 T-cell differentiation - Impedes B-cell responses - Provokes apoptosis via Fas/FasL pathway	High	High	High	(29, 30, 105, 109, 110)
Memory	- Trigger low expression of effecter molecules - Arouses the low expression of pro-inflammatory cytokines	Intermediate	Intermediate	Intermediate	(29, 105, 106, 110)
**CD8**^**+**^**T-cells**					
Naïve	- Proliferates CD8^+^ T-cells in absence of antigens	Low	Intermediate	Low	(29, 33, 109)
Activated	- Induces the proliferation of antigen-specific clones - Supports effector differentiation. - Enhances (other) pro-inflammatory cytokine secretion (*e.g.,* IFN-γ, TNF-α and LT-α) - Promotes cytolytic activity and cell killing via perforins and granzymes	High	High	High	(29, 30, 32, 105, 110)
Memory	- Proliferates memory CD8^+^ T-cells - Trigger low expression of effecter molecules - Arouses the low expression of pro-inflammatory cytokines	Low	High	Low	(29, 30, 105)
**T**_**reg**_**-cells**					
Naïve	- Crucial for T_reg_ development in the thymus - Maintains thymus and peripheral T_reg_ in secondary lymphoid tissues - Activates T_reg_ when bound to dendritic cells	High	High	High	(29, 105–108, 111, 112)
Activated	- Expandss and differentiates T_reg_ in peripheral tissues - Stimulates the T-cell proliferation inhibition - Encourages the T-cell secretion of immunosuppressive cytokines (*e.g.,* IL-9, IL-10 and TGF-β) - Supports survival and lineage stability - Maintains glycolysis	High	High	High	(105, 107, 109, 112–114)
Memory	- Generates memory T_reg_ from naïve CD4^+^ T-cells	High	Unknown	Unknown	(113)
**NK-cells**					
Naïve	- Proliferates NK-cells - Activates NK-cells	High*	High**	High***	(115–117)
Activated	- Proliferates NK-cells - Differentiates NK-cells - Promotes cytolytic activity - Enhances (other) cytokine production	High	High	High	(30, 116–118)

Abbreviations: interleukin (IL-), interferon gamma (IFN-γ), tumor necrosis factor-alpha (TNF-α), lymphotoxin-alpha (LT-α), regulatory T-cells (T_reg_), transforming growth factor-beta (TGF-β), natural killer cells (NK-cells). * 5% of naïve NK-cells express IL-2Rα. ** 21% of naïve NK-cells express IL-2Rβ. *** 22% of naïve NK-cells express IL-2.

One of the major IL-2 activities is the stimulation of T-cell proliferation and effector T-cell (T_eff_) differentiation, *i.e.*, the generation of immunity [30]. The differentiation towards distinct effector CD4^+^ T-cell subsets is clearly reviewed by Klatzmann and Abbas (2015) [31]. However, IL-2 is also involved in (self-)tolerance and homeostasis by both induction and survival of T_reg_, as well as activation-induced cell death [29,31]. The paradoxical function in immunity and tolerance is best illustrated in the experiments performed by Knoechel et al. (2005) [2]. In a murine model for autoimmune disease, T_eff_ amplification was associated with onset of clinical manifestation, while T_reg_ expansion preceded the recovery phase. In the absence of IL-2, the onset of disease was delayed, but animals did not recover and even succumbed due to a very severe disease. This phenomenon was attributed to the reduced proliferation and differentiation of T_eff_ in combination with a complete lack of T_reg_ development. Another major activity of IL-2 is the regulation of key aspects of CD8^+^ T-cells such as cell differentiation towards memory versus effector CD8^+^ T-cells [32]. Data suggest that the level of IL-2 signalling determines the shift towards memory versus effector CD8^+^ T-cells [33,34]. Kalia et al. (2010) demonstrated that low level of IL-2 signalling differentiated CD8^+^ T-cells into functional long-lived memory cells and upregulated CD127 and CD62L phenotypes. On the other hand, high level of IL-2 signalling resulted in effector CD8^+^ cell differentiation, the down-regulation of the memory cell phenotypes CD127 and CD62L and the upregulation of Blimp-1 [33]. Subsequently, Blimp-1 suppressed IL-2 production, forming a negative feedback loop [35,36]. In addition to the IL-2 interaction with T-cells, IL-2 also impacts NK-cells. Previous reviews have clearly explained that IL-2 is capable to differentiate, proliferate and activate NK-cells [[37], [38], [39]].

**Fig. 1:**
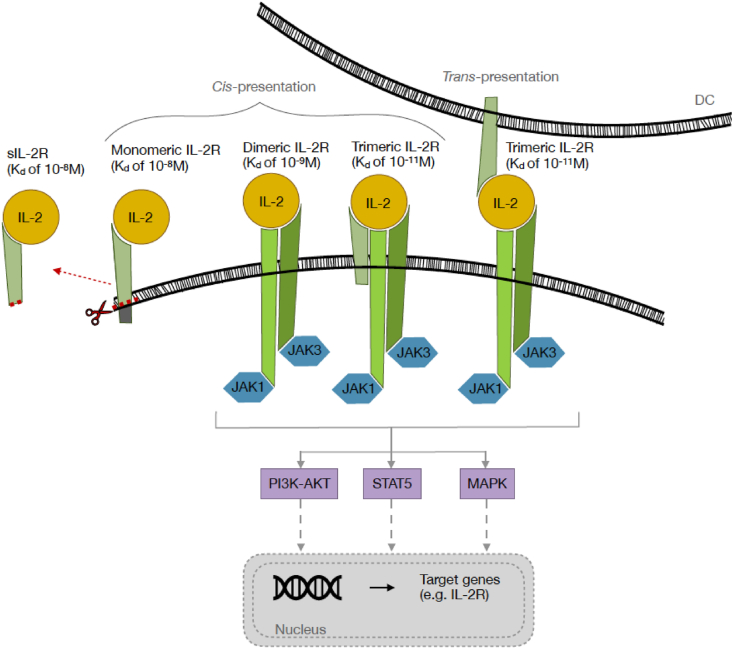
**The IL-2R classification and the IL-2 – IL-2R signalling pathway.** IL-2 binds to the monomeric (low binding affinity: K_d_ of ∼10^−8^ M), dimeric (intermediate binding affinity: K_d_ of ∼10^−9^ M), trimeric (high binding affinity: K_d_ of ∼10^−11^ M) and soluble (binding affinity: K_d_ of ∼10^−10^ M, sIL-2R) IL-2R. The monomeric IL-2R consists of only the IL-2Rα-chain. The heterodimeric receptor is composed of the combination of the IL-2Rβ and -γ forms, while the trimeric receptor combines all three subunits. The trimeric IL-2R can be constructed by the *cis*-presentation of IL-2Rα on T-cells and the *trans*-presentation of IL-2Rα on antigen presenting cells (*e.g.,* dendritic cells, DCs). The IL-2Rα-chain can be shed off, generating the sIL-2R. Only the binding of IL-2 to the dimeric and trimeric IL-2Rs results in downstream signalling via three main pathways: PI3K-AKT, STAT5 and MAPK. These pathways activate the transcription of target genes such as the IL2RA gene.

IL-2 exerts its effect via binding to the IL-2R and subsequent intracellular signalling (Fig. 1). Since the molecular structure, multimeric composition and downstream signalling of IL-2R has been extensively reviewed elsewhere [40,41], this will only be summarized in the current review. Three proteins may be differentially involved in the functional IL-2R complex: the IL-2Rα-chain (CD25 or Tac; 55 kDa), the IL-2Rβ-chain (CD122; 75 kDa), and the IL-2Rγ-chain (CD132 or common γ (γ_c_)-chain; 64 kDa). The IL-2R may consist of a monomeric IL-2Rα-chain, a heterodimeric receptor composed of the IL-2Rβ- and γ-chain, and a trimeric receptor composed of all three subunits. While the monomeric IL-2R only has low affinity for IL-2 (K_d_ of ∼10^−8^ M), the dimeric and trimeric receptors have intermediate and high affinity (K_d_ of ∼10^−9^ M and ∼10^−11^ M), respectively. Of note, the dimeric receptor is not specific for IL-2, but also can bind IL-15. Moreover, the IL-2Rγ-chain is also part of the distinct receptors for IL-4, IL-7, IL-9, IL-15, and IL-21, hence the alternative name of common γ-chain. The distinct compositions of the IL-2R are differentially expressed on T-cells and NK-cells (Table 1). Downstream signalling only occurs via the IL-2Rβ- and γ-chain, but not the α-chain because the latter lacks an intracellular domain. However, the α-chain is important for an additive effect (increasing the binding affinity) on IL-2 binding which may occur via *cis*-presentation in the trimeric receptor or *trans*-presentation to the dimeric receptor [40,41].

Interaction of IL-2 with the IL-2R results in oligomerization and conformation changes in the IL-2R subunits, eventually resulting in downstream signalling. This starts with phosphorylation of the intracellular domains of Janus tyrosine kinase (JAK)1 and JAK3, attached to IL-2Rβ and IL-2Rγ chains, respectively. Next, distinct tyrosine residues in the cytoplasmic tail of the IL-2Rβ chain are phosphorylated resulting in further downstream events. The three main downstream-pathways involve signal transducer and activation of transcription (STAT), phosphoinositide-3-kinase (PI3K)- protein kinase B (AKT) and mitogen-activated protein kinase (MAPK), eventually resulting in the transcription of multiple target genes. The evoked transcription factors differentially activate the genes encoding for IL-2 and IL-2R chains [40,41]. After binding of IL-2 to the IL-2R, the receptor is ingested into endosomes. In T-cells the IL-2Rα-chain is present in the recycling endosomes (transferrin-positive) while the IL-2Rβ and γ chains are only found in the late endosomes (rab7-positive). This illustrates that IL-2Rα is disconnected from IL-2Rβγ. In addition, IL-2Rα will be recycled to the cell surface whereas IL-2Rβγ will be degraded [42,43]. The timing and functional effect of this phenomenon remains to be elucidated.

Recycling IL-2Rα may be related to the fact that IL-2Rα can be shed off from the cell surface of activated human immune cells (T-, B-, and malignant cells, and DCs, monocytes, and macrophages), forming the sIL-2R (Fig. 1) [3]. sIL-2R is a 45 kDa protein instead of the 55 kDa membranous equivalent due to the absent intra-cytoplasmic and transmembrane regions. Despite of this loss, sIL-2R has a similar capability to bind IL-2 as the membrane bound IL-2Rα with an affinity of K_d_ of ∼10^−8^ M [44,45]. However, the underlying mechanism of shedding off the membrane-bound IL-2Rα remains to be elucidated. Potential mechanisms are cell death [46], proteolytic cleavage [47], genes encoding for the soluble and cellular counterparts of IL-2Rα [48] and alternative splicing of receptor mRNA [47]. Also, the function of sIL-2R has to be defined yet, although there are some theories about it: repressing immunity [49,50], supporting immunity [[51], [52], [53]] or no influence on immunity [54] via capturing IL-2 [55], extending IL-2's half-life [56] or changing IL-2's structure within helix C and thereby augmenting the binding affinity between IL-2 and IL-2Rβγ [57].

## 4 The IL-2 – IL-2R pathway in multiple sclerosis

In this paragraph the different components of the IL-2 – IL-2R pathway will be discussed in relation to susceptibility to and progression of MS. This involves potential genetic variants and their effect on protein expression and function (Table 2). Finally, functional implications of sIL-2R will be discussed in the context of potential mechanisms relevant for MS.

**Table 2:** The genetic and protein relation of IL-2 (A), IL-2R (B) and sIL-2R (C) in MS.

**A**	**IL-2**		
**Genetic level**	**Ref.**	**Protein level**	**Ref.**
- Rs2069762 and rs2069763 polymorphisms are recognized as *IL2* risk-gene for MS, although dependent on ethnicity.	(58–62)	- RRMS and SPMS patients have increased IL-2 serum and CSF levels.	(66, 67)
- The reduced frequency of the rs2069762 GT- and TT-genotype is related to SPMS, but not RRMS, in Caucasian individuals.	(59)	- American healthy individuals with the rs2069762 GG-genotype have augmented IL-2 production by activated T-cells.	(68)
- Iraqi individuals are more susceptible to MS when a diminished T-allele frequency in the rs2069763 is present, while no susceptibility to MS was observed when carrying rs2069762.	(62)	- Iranian and Egyptian MS patients with the rs2069762 TT-genotype have elevated IL-2 levels, compared to healthy controls and MS patients with the GG- or GT-genotype.	(69, 70)
- Japanese and Iranian populations, carrying rs2069762 and rs2069763, are not related to the susceptibility of MS.	(60, 61)	- The frequency of circulating IL-2 secreting cells is higher in MS than OND-patients	(71)
- *IL2* polymorphisms have not been identified as risk factor for MS in GWAS.	(63–65)	- No differences are obtained in MS patients in relation to disease activity and/or duration of disease.	(71)
**B**	**IL-2R**		
**Genetic level**	**Ref.**	**Protein level**	**Ref.**
- *IL2RA* is recognized as a risk gene for MS in GWAS while *IL2RB* and *IL2RG* are not.	(65, 72)	- Individuals carrying rs2104286 AA-genotype have lower IL-2Rα expression on peripherally expanded T_h_-cells (including subsets of conventional T-cells and T_reg_) and higher IL-2Rα expression on recent thymic emigrants and naïve T_reg_.	(76, 77)
- Risk *IL2RA* polymorphisms include rs2104286, rs12722489, rs3118470, rs1570538 and rs11594656	(65, 73–75)	- Diminished IL-2Rα expression on cells seems not to be related to lower IL-2 responses but to additional factors.	(77)
- Rs2104286 A-haplotype, rs12722489 C-haplotype, rs3118470 G-haplotype as well as rs1570538 T-haplotype and/or rs11594656 A-haplotype showed a predisposition to MS in Caucasian individuals.	(65, 73–75)	- Rs2104286 A-haplotype is associated with lower IL-2R signalling in CD45^+^CD25^hi^ T-cells and increased production of GM-CSF in memory T_h_-cells.	(76–78)
- Caucasian individuals carrying rs1570538 TT-genotype have a further increased risk to develop RRMS, compared to the CC-genotype.	(73)	- Rs2104286 G-haplotype is related to decreased frequency of CD25^+^ naive T-cells.	(79)
- Asian individuals are more susceptible for MS when carrying rs2104286 A-haplotype.	(74)	- Rs2104286 A- and G-haplotype are associated with altered *IL2RA* transcription factor binding and activity.	(80)
- The *IL2RA* promotor region is susceptible to epigenetic regulation	(85)	**-** CD56^bright^ NK/IL-17 A^+^CD4^+^ T-cell ratio is lower in rs3118470 G-haplotype carriers despite that no correlation was found between total *IL2RA* gene expression levels and NK/T-cell ratio.	(81)
		- MS patients have more IL-2R expressing cells in affected brain tissue.	(84)
**C**	**sIL-2R**		
**Genetic level**	**Ref.**	**Protein level**	**Ref.**
**The same as *IL2RA (section B)***		- MS patients have augmented sIL-2R levels in serum and CSF, especially when having relapses.	(86–88)
		- No significant alterations are found among the MS disease courses CIS, RRMS, SPMS and PPMS.	(88, 90)
		- Contradictory data about the stability of sIL-2R levels in MS and healthy individuals.	(6, 81, 88, 89)
		- RRMS patients with a malignant disease course, compared to a benign disease course, have elevated sIL-2R levels.	(88)
		- Increased sIL-2R production associate with rs2104286 (A-haplotype), rs11594656 (A-haplotype) and rs3118470 (G-haplotype).	(75, 81, 90)
		- sIL-2R seems to function as an IL-2 antagonist by down-regulating pSTAT5 in T_h_-cells, but also as a decoy receptor.	(77, 88, 91)
		- Patients with follicular lymphoma showed augmented pSTAT5 in T_h_-cells, but sIL-2R shows conflicting roles in health and diseases.	(92, 93)
		- Subcutaneous sIL-2R administration in EAE models resulted in exacerbated disease.	(91)
		- The effect of sIL-2R on T-cell (subsets) proliferation are inconsistent.	(93)

Abbreviations: interleukin 2 (IL-2); IL-2 receptor (IL-2R); soluble IL-2R (sIL-2R); multiple sclerosis (MS); relapsing and remitting MS (RRMS); secondary progressive MS (SPMS); genome wide association studies (GWAS); cerebrospinal fluid (CSF); helper T-cells (T_h_); regulatory T-cells (T_reg_); granulocyte-macrophage colony stimulating factor (GM-CSF); clinically isolated syndrome (CIS); primary progressive MS (PPMS); natural killer cells (NK-cells); phosphorylation of signal transducer and activation of transcription 5 (pSTAT5); experimental autoimmune encephalomyelitis (EAE).

### 4.1 Interleukin-2

In humans, the IL-2 gene (*IL2*) is encoded on chromosome 4 section q26-q27, containing four exons. Two genetic polymorphisms have been investigated in terms of predisposition to MS: rs2069762 and rs2069763 [58]. The rs2069762 is located within the promoter region, while rs2069763 is a silent mutation located in exon 1. Matesanz (2001) reported a reduced frequency of the GT- and TT-genotype of rs2069762 in Caucasian SPMS, but not RRMS patients [59]. Subsequent studies in Japanese, Iranian and Iraqi populations could not find any association between this polymorphism and MS [[60], [61], [62]]. With respect to rs2069763, only the Iraqi study observed a lower frequency of the T-allele in MS patients as compared to healthy controls, while the other three studies did not find any association [[59], [60], [61], [62]]. Overall, rs2069762 might be a risk factor for progression to SPMS in Caucasian MS patients, while rs2069763 might be a risk factor for susceptibility to MS in Iraqi MS patients, but not in patients of other ethnicities. Importantly, polymorphisms in *IL2* have not been identified as risk factor for MS in genome wide association studies (GWAS) [[63], [64], [65]].

Previous studies demonstrated augmented IL-2 levels in serum and CSF of RRMS and SPMS patients, compared to individuals with other neurological diseases (OND) and normal controls [66,67]. Since rs2069762 is located within the promoter region, the genetic variants may influence IL-2 gene expression. Indeed, the IL-2 production by activated T-cells was increased in American healthy controls carrying the GG-genotype [68]. However, Iranian and Egyptian MS patients carrying the TT-genotype had higher circulating IL-2 levels than both healthy controls with the TT-genotype, as well as MS patients with the GG- or GT-genotype [69,70]. While differential IL-2 levels in the circulation may be due to genetic differences, this could also be explained by different frequencies of IL-2 producing cells. Indeed, MS patients have higher circulating numbers of IL-2 secreting cells as compared to patients with OND [71]. No differences were observed within MS patients in relation to disease activity and/or duration of disease [71]. Overall, IL-2 levels are increased in MS patients, but this seems less to be related to genetic polymorphisms in the *IL2*-gene and more by a difference in frequency of IL-2 producing cells.

### 4.2 Interleukin-2 receptor

The human genes encoding IL-2Rα (*IL2RA),* IL-2Rβ (*IL2RB*) and IL-2Rγ (*IL2RG*) are located on chromosome 10 region p15.1, chromosome 22 region q12.3 and chromosome X region q13.1, respectively. GWAS recognized *IL2RA* as a risk gene for MS (odds ratio 1.1–1.3), but not *IL2RB* and *IL2RG* [65,72]. Therefore, we focussed on genetic polymorphisms only in the *IL2RA*. These include, among others, intron variants rs2104286, rs12722489, and rs3118470, the 3′ prime UTR variant rs1570538 and the *IL2RA* 5’ region variant rs11594656 [65,[73], [74], [75]]. Caucasian individuals are more susceptible for MS (odds ratio ∼1.2) when carrying either the rs2104286 A-haplotype, rs12722489 C-haplotype, rs3118470 G-haplotype, rs1570538 T-haplotype and/or rs11594656 A-haplotype, whereas Asian individuals only have elevated risk for MS when carrying the rs2104286 A-haplotype (odds ratio 1.25) [65,[73], [74], [75]]. In addition, Caucasian individuals carrying the rs1570538 TT-genotype (odds ratio 1.69) showed a further increased risk for RRMS as compared to carriers of the CC-genotype [73].

Some studies have reported associations of *IL2RA* polymorphisms with differences in the level of IL-2Rα expression [76,77]. The rs2194286 AA-genotype was associated with reduced IL-2Rα expression in peripherally expanded T_h_-subsets, including different types of conventional T-cells and T_reg_. Of note, although the reduced IL-2Rα expression levels on T-cells could be attributed to a diminished response to IL-2, additional factors seem to be involved [77]. Recent thymic emigrants and naïve T_reg_, on the other hand, showed increased IL-2Rα expression in association with the rs2104286 AA-genotype [76,77].

Other studies, have reported functional associations of *IL2RA* polymorphisms. The rs2104286 A-haplotype is associated with lower IL-2R signalling in CD4^+^CD25^hi^ T-cells [76,77] and augmented production of granulocyte-macrophage colony stimulating factor (GM-CSF) in memory T_h_-cells [76,78]. The rs2104286 G-haplotype is related to reduced frequency of CD25^+^ naïve T-cells [79] and both rs2104286 A- and G-haplotypes are associated with influenced *IL2RA* transcription factor binding and activity [80]. Finally, the rs3118470 risk allele (G) is related to a diminished CD56^bright^ NK/IL-17 A^+^CD4^+^ T-cell ratio in MS patients, although *IL2RA* gene expression levels do not correlate with NK/T-cell ratios [81].

Altogether, the genetic polymorphisms in the *IL2RA* gene seem to have an effect on the expression and function of the IL-2R, but it should be taken into account that also other factors will influence gene expression [82,83]. Indeed, some studies have reported more IL-2R expression in the brain of MS patients as compared to controls [84]. This more likely represents a difference in leukocyte infiltration and local activation instead of a differential regulation of IL-2R expression. Furthermore, the promoter region of the *IL2RA* gene is susceptible to epigenetic regulation [85]. Importantly, this epigenetic regulation is independent from the genetic polymorphisms identified.

### 4.3 Soluble interleukin-2 receptor

Several studies investigated sIL-2R levels in MS patients. Adachi et al. (1990) demonstrated that MS patients had significantly higher sIL-2R serum and CSF levels during a relapse as compared to both remission as well as controls [86]. The control group for serum and CSF levels consisted of healthy and OND individuals, respectively. Sharief and Thompson (1993) confirmed that sIL-2R CSF levels were significantly elevated in relapse patients compared to remission and OND individuals [87]. However, with respect to sIL-2R serum levels only a trend towards higher levels in relapse versus remission patients was observed. Next, Maier et al. (2009) confirmed elevated sIL-2R serum levels in RRMS patients compared to healthy controls [88]. Furthermore, sIL-2R serum levels appeared rather stable, both in MS patients as well as in healthy controls. In the placebo group of the SOLARIUM study, serum sIL-2R levels at week 0 and week 48 correlated very strongly [6,81], although the median levels tended to slightly decrease over time [6,81]. However, Freedman et al. (1992) determined sIL-2R serum levels with shorter intervals and this revealed that there can be substantial variation over time in both RRMS patients and healthy controls [89]. In MS patients there was some correlation with clinical relapses, but the potential predictive value for clinical disease activity was limited. Finally, Maier et al. (2009) and Buhelt et al. (2017) measured the sIL-2R serum levels in CIS, RRMS, SPMS and PPMS patients, but this resulted in no significant differences among the groups [88,90]. However, RRMS patients with a malignant disease course, as defined by a fast progression of the expanded disability status scale (EDSS) score over time, had higher sIL-2R levels as compared to RRMS patients with a benign disease course [88]. Since sIL-2R basically is an expression product of the *IL2RA* gene, several studies have investigated the effect of *IL2RA* polymorphisms on serum sIL-2R levels. Higher sIL-2R levels were observed in individuals with the rs2104286 A-haplotype, rs11594656 A-haplotype and rs3118470 G-haplotype [75,81,90]. As described above, these alleles have been associated with susceptibility to MS.

Studies investigating the potential role of sIL-2R in the pathogenesis of MS are lacking. Functional studies on the effect of sIL-2R on T-cells of human healthy controls have been performed by Maier et al. (2009) [88]. As expected, both conventional (CD4^+^FOXP^−^) and regulatory T_h_-cells (CD4^+^FOXP^+^) showed STAT5 phosphorylation (pSTAT5) upon incubation with IL-2. Addition of sIL-2R resulted in a dose-dependent down-regulation of pSTAT5. Although the effect was less pronounced in T_reg_ as compared to conventional T_h_-cells, it was concluded that sIL-2R acts as an antagonist in the IL-2 – IL-2R pathway. This is in line with the observation of Cerosaletti et al. (2013), demonstrating that the percentage of pSTAT5-positive T_reg_ (CD4^+^CD25^+^) after *ex vivo* activation with IL-2, negatively correlated with serum sIL-2R levels in healthy individuals [77]. In addition, Russell et al. (2012) showed that sIL-2R inhibits pSTAT5 in murine T_h_17-cells [91]. They demonstrated that subcutaneous administration of sIL-2R in the murine EAE model resulted in exacerbated disease. It was concluded that sIL-2R acts as a decoy receptor, and as such causes decreased signalling downstream of the IL-2R, eventually enabling expansion of T_h_17-cells and T_h_1-cells. In contrast to the studies mentioned above, Yang et al. (2011) showed that sIL-2R enhanced pSTAT5 in CD4^+^ T-cells from patients with follicular lymphoma [92]. This controversial result may be caused due to the inclusion of T-cells from patients, since the roles of sIL-2R are conflicting in health and diseases [93]. Whether this also holds for MS remains to be determined, and it is tempting to speculate that this is linked to the functional loss of CD4^+^CD25^+^ T_reg_ in MS patients [22]. Besides effects on pSTAT5, several studies have investigated the effect of sIL-2R on proliferation of T-cells and T-cell subsets of healthy controls. These studies also revealed inconsistent results (reviewed in Ref. [93]).

Taken together, sIL-2R levels are elevated in serum and CSF of MS patients, but do not differentiate between disease subtypes. Nevertheless, higher levels were prognostic for a malignant course of disease, but intermittent increases have limited predictive value for clinical disease activity. Functional implications of increased sIL-2R levels in MS patients need to be further defined. There are indications that sIL-2R acts as an antagonist of the IL-2 – IL-2R pathway, possibly as a decoy IL-2R, eventually disturbing the balance in the T_h_-cell compartment. These mechanisms have been investigated *in vitro* with human PBMC or in the EAE model. Further support for the role of sIL-2R in the pathogenesis of MS remains to be elucidated.

## 5 Discussion

It is evident that the IL-2 – IL-2R pathway is involved in the balance between immunity and tolerance. Components of this pathway are associated with both development as well as progression of multiple autoimmune diseases, including MS. First, the *IL2RA* gene has been identified in GWAS as a risk gene for MS [65,72]; polymorphisms in this gene are associated with the level of expression of the IL-2Rα-chain on immune cells [76,77]. Second, levels of IL-2 are increased both in serum as well as CSF. Furthermore, MS patients have more IL-2R expressing cells in affected brain tissue. Although these findings support the role of the IL-2 – IL-2R pathway in MS, the increased levels of the respective parameters more likely represent immune activation during disease progression instead of an intrinsic default mechanism causal to the development of MS. Third, MS patients have increased levels of sIL-2R, especially during active disease [[86], [87], [88]], but the role of this molecule is only poorly defined. In order to illustrate the complexity of the IL-2 – IL-2R pathway, we will further discuss on the role of this pathway, and in particular of sIL-2R, in the context of environmental factors and therapeutic approaches by the examples of vitamin D and daclizumab, respectively.

Vitamin D deficiency is a well-recognized risk factor for MS and may also influence disease progression [94,95]. Interestingly, vitamin D is directly linked with the IL-2 – IL-2R pathway, and in particular with the expression of the IL-2Rα-chain. As such, vitamin D is considered a modulator in the physiologic regulation of T-cell homeostasis [7]. The *IL2RA* gene expression, as well as the IL-2Rα cell surface expression, is increased on *in vitro* activated CD4^+^ T-cells upon incubation with 1,25(OH)_2_D_3_, the active component of vitamin D [83]. These data are further supported by the observed positive association between circulating 25 (OH)D levels and the expression of the *IL2RA* gene. Differentiated analyses of conventional CD4^+^ T-cells and T_regs_, derived from MS patients supplemented with vitamin D, did not confirm increased surface expression of the IL-2Rα-chain [6]. However, vitamin D supplemented MS patients displayed a loss of the IL-2Rα-chain over time, to which T_regs_ contributed most within the CD4^+^ T-cell fraction. Apparently, the effects of vitamin D supplementation are different for distinct T-cell subsets and, indeed, this is further supported by the studies of Killick et al. (2020) [96]. In a more complicated experiment with *in vitro* activated CD4^+^ T-cells from MS patients supplemented with vitamin D or placebo, cluster analyses were performed on the phenotypic charactistics of CD4^+^ T-cells. Subsets that increased in relative proportion upon vitamin D supplementation had lower cell surface IL-2Rα-chain expression, while subsets that decreased had higher levels of expression. Rolf et al. (2018) also investigated whether the altered IL-2Rα-chain expression upon vitamin D supplementation affects circulating sIL-2R levels [6]. Data reveal that sIL-2R levels typically decline in the placebo group, while sIL-2R levels remain rather stable in the vitamin D group. Based on these data it was speculated that vitamin D may interfere with the apparently dynamic complex network of the IL-2 – IL-2R pathway and, hereby, may better preserve the delicate balance within the immune system.

Within the broad spectrum of therapies for MS daclizumab, an anti-IL-2Rα humanized monoclonal antibody, directly interferes with the IL-2 – IL-2R pathway [97,98]. By blocking IL-2Rα it was intended to disrupt the ongoing activation of autoreactive T-cells [99]. However, daclizumab simultaneously will inhibit the function of T_reg_ and thereby may counteract the effect on autoreactive T-cells. Indeed, studies revealed that daclizumab stimulated T-cell responses, indicating that daclizumab has a pro-inflammatory activity [8,100]. In light of the positive effect of daclizumab on disease activity, an alternative mechanism of action was discovered by the observation that treatment resulted in a very strong expansion of NK-cells [8,101]. The expansion involved CD56^bright^ NK-cells, expressing the IL-2Rβγ. Due to blocking the IL-2Rα-chain, increased amounts of IL-2 were available for cells expressing the intermediate affinity IL-2R, eventually resulting in excessive proliferation of NK-cells. These CD56^bright^ NK-cells appeared to be cytotoxic for autoreactive CD4 T-cells [8,102]. This illustrates the immunomodulatory function of NK-cells in MS and the relevant interplay between T- and NK-cells due to the possible mediatory role of IL-2R. These findings have increased the awareness that, next to T- and B-cells, also NK-cells seem to be a relevant player in the pathogenesis of MS [10]. Mimpen et al. (2021) demonstrated that NK-/T-cell ratios may be a prognostic biomarker for MS disease activity [103]. MS patients with MRI-activity or relapses at week 48 had lower NK-/IL-17 A^+^CD4^+^ T-cell ratios at baseline. In addition, NK-/IL-17 A^+^CD4^+^ T-cell ratios correlated negatively with neurofilament chain levels. Ongoing studies revealed an association between NK-/IL-17 A^+^CD4^+^ T-cell ratios and sIL-2R protein shedding (*in vitro* and *in vivo*), as well as with IL-2Rα protein expression on CD4^+^ T-cells [81]. Furthermore, higher serum sIL-2R levels and lower CD56^bright^ NK-/IL-17 + CD4^+^ T-cell ratios correlated with the rs3118470 risk allele. These studies support a role of the IL-2 – IL-2R pathway in establishing the NK-/T-cell ratio, further emphasizing the importance of this pathway in the disease course of MS.

**Fig. 2:**
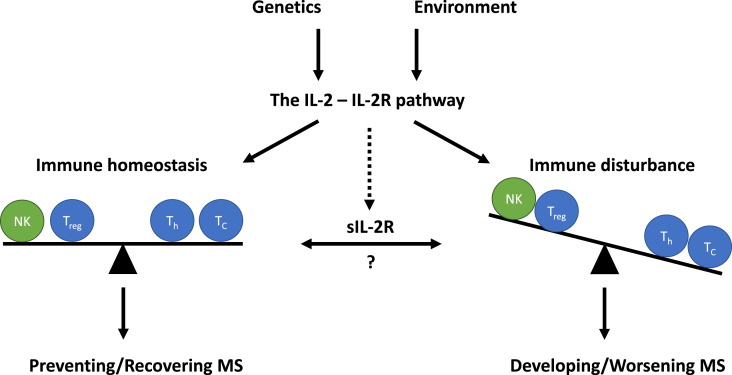
**The interleukin (IL-)2 – IL-2 receptor (IL-2R) pathway in multiple sclerosis (MS).** The role of the IL-2 – IL-2R pathway in MS is influenced by genetic and environmental factors. This can lead to immune homeostasis (balance of regulatory, helper and cytotoxic T-cells (T_reg_, T_h_ and T_c_, respectively) and natural killer cells (NK-cells)) or immune disturbance, less NK-cells and T_reg_ and more T_h_- and T_c_-cells. Subsequently, a balanced immune homeostasis enables prevention and recovering of MS, while disturbed immune responses enable development and worsening of MS. Furthermore, the soluble form of the IL-2R (sIL-2R) also seems to play an important role within these outcomes. However, the exact function remains to be elucidated.

Overall, it is evident that MS is a complex disease that develops and evolves by an interplay between genetic and environmental factors resulting in a disturbed immune homeostasis affecting multiple types of immune cells. The IL-2 – IL-2R pathway has been identified as a crucial factor to maintain immune homeostasis and, therefore, might be an interesting pathway for therapeutic intervention (Fig. 2). However, as exemplified by daclizumab, IL-2 appears to have a dual and opposing function. This is further complicated by the lack of understanding the exact role of sIL-2R. While sIL-2R may function as an IL-2 agonist or antagonist, this is to be interpreted differently in the context of conventional T-cells or T_reg_. Furthermore, it is to be expected that daclizumab also binds to sIL-2R, suggesting that the treatment outcome may be different in MS patients with high levels versus low levels of sIL-2R. Altogether, it will be a challenge to manipulate the IL-2 – IL-2R pathway, including sIL-2R levels, to restore the balance between immunity and tolerance. Strategies based on IL-2 and/or IL-2R variants may selectively target distinct cell types, while keeping the function of other cells unchanged [104]. Further research on this intriguing pathway is warranted and key to understanding MS.

## Formatting of funding sources

This research did not receive any specific grant from funding agencies in the public, commercial, or not-for-profit sectors.

## Declaration of competing interest

The authors declare that they have no known competing financial interests or personal relationships that could have appeared to influence the work reported in this paper.
